# Potential Geographic Distribution of Hantavirus Reservoirs in Brazil

**DOI:** 10.1371/journal.pone.0085137

**Published:** 2013-12-31

**Authors:** Stefan Vilges de Oliveira, Luis E. Escobar, A. Townsend Peterson, Rodrigo Gurgel-Gonçalves

**Affiliations:** 1 Postgraduate Program in Tropical Medicine, Universidade de Brasília, Brasília, Brazil; 2 Unidade Técnica de Vigilância de Zoonoses, Ministério da Saúde, Brasília, Brazil; 3 Conservation Medicine Program, Facultad de Ecología y Recursos Naturales, Universidad Andres Bello, República 440, Santiago, Chile; 4 Biodiversity Institute, University of Kansas, Lawrence, Kansas, United States of America; 5 Laboratório de Parasitologia Médica e Biologia de Vetores, Universidade de Brasília, Brasília, Brazil; Fondazione IRCCS Policlinico San Matteo, Italy

## Abstract

Hantavirus cardiopulmonary syndrome is an emerging zoonosis in Brazil. Human infections occur via inhalation of aerosolized viral particles from excreta of infected wild rodents. *Necromys lasiurus* and *Oligoryzomys nigripes* appear to be the main reservoirs of hantavirus in the Atlantic Forest and Cerrado biomes. We estimated and compared ecological niches of the two rodent species, and analyzed environmental factors influencing their occurrence, to understand the geography of hantavirus transmission. *N. lasiurus* showed a wide potential distribution in Brazil, in the Cerrado, Caatinga, and Atlantic Forest biomes. Highest climate suitability for *O. nigripes* was observed along the Brazilian Atlantic coast. Maximum temperature in the warmest months and annual precipitation were the variables that most influence the distributions of *N. lasiurus* and *O. nigripes*, respectively. Models based on occurrences of infected rodents estimated a broader area of risk for hantavirus transmission in southeastern and southern Brazil, coinciding with the distribution of human cases of hantavirus cardiopulmonary syndrome. We found no demonstrable environmental differences among occurrence sites for the rodents and for human cases of hantavirus. However, areas of northern and northeastern Brazil are also apparently suitable for the two species, without broad coincidence with human cases. Modeling of niches and distributions of rodent reservoirs indicates potential for transmission of hantavirus across virtually all of Brazil outside the Amazon Basin.

## Introduction

Hantaviruses (family *Bunyaviridae*, genus *Hantavirus*) are zoonoses that have been appreciated increasingly in the last two decades, particularly across the Americas. Human infections occur by inhalation of aerosolized viral particles from excreta of infected wild rodents. The disease manifests in different clinical forms: hemorrhagic fever with renal syndrome (HFRS) and hantavirus cardiopulmonary syndrome (HCPS); HCPS is an emerging disease, particularly in the Americas [1]. The first documented cases of HCPS emerged apparently as a result of weather phenomena that favored exposure of human populations to wild rodents [2,3]. 

In Brazil, the first recorded occurrence of HCPS was in 1993, in the city of Juquitiba, state of São Paulo [4]. Since then, knowledge of the disease has expanded and its range is now known to extend to all regions of the country [5]. Among rodent species found infected with hantavirus in Brazil, *Necromys lasiurus* and *Oligoryzomys nigripes* are widely distributed in the Cerrado and Atlantic Forest biomes, respectively [6,7]. Some studies indicate that regional distributions of hosts and pathogen are related to climatic and environmental factors [8-11]; these studies also show associations between occurrence of human cases of hantavirus and areas of high probability of occurrence of rodent reservoirs, suggesting that analysis of ecological niche models of hosts could offer valuable information. Therefore, assessment of potential distributions of rodent reservoirs in different biomes and analysis of the influence of environmental factors on hantavirus transmission can be useful in understanding spatial patterns of transmission risk of the disease. 

This study explores a series of approaches. We estimated ecological niches and geographic distributions for *N. lasiurus* and *O. nigripes* in the Cerrado and Atlantic Forest regions, and analyzed environmental factors associated with their occurrence. We estimated the ecological niche for hantavirus transmission in Brazil based on the distribution of infected rodents detected during 2000-2010. Finally, we compared distributions with respect to climatic conditions for the two rodent species and for HPCS cases in humans, to ascertain whether they occur under distinct environmental circumstances. 

## Materials and Methods

### Occurrence data

All data were obtained from public databases; full names and information for accessing data (websites) are provided below. All data were anonymized. Distributional data for *N. lasiurus* and *O. nigripes* were obtained from speciesLink (http://splink.cria.org.br), Global Biodiversity Information Facility (http://data.gbif.org) and VertNet (http://www.vertnet.org); our searches included the old name of *N. lasiurus* (*Bolomys lasiurus*). We also consulted various published studies on these rodent species [12-33]. When only textual georeferences were provided we georeferenced them based on two internet resources (http://www.fallingrain.com/world/ and http:// www.ibge.gov.br/). Records were georeferenced with an uncertainty of ≤5 km to the nearest 0.01° (records presenting greater uncertainty were removed). We eliminated duplicate records and records presenting obvious errors of georeferencing or identification (e.g., records in the ocean). We also excluded records in oversampled locations based on subsampling among very close pairs of points to reduce sampling bias [34]. Based on occurrence data, we created buffers of 400 km around known occurrences, which were used as a hypothesis of the accessible area **M** [35] which is the area most appropriately used for model calibration.

For information on rodents infected with hantavirus, we used technical reports of research activities undertaken by the Brazilian Ministry of Health. We also extracted occurrence data from published records of infections in these two rodent species during 2000-2010 [7,30,36-48].

Finally, we included records of HCPS cases reported from municipalities falling within the bounds of the Atlantic Forest and Cerrado biomes, reported by the Brazilian Ministry of Health during 2000-2010. We considered in particular suspected infection location, where human infection by the pathogen apparently occurred. Locations for infected rodents and humans were georeferenced as described above.

### Environmental data

To characterize environmental variation across Brazil, we used seven climatic variables: annual mean temperature, diurnal temperature range, maximum temperature in the warmest month, minimum temperature in the coldest month, annual precipitation and precipitation in the wettest and driest months. We obtained these variables from the WorldClim project (worldclim.org), which were developed via interpolation of mean monthly climatic data from meteorological stations over 30-50 (1950-2000) years, depending on data availability at stations [49]. To summarize aspects of vegetation and land cover, we used multitemporal (monthly) normalized difference vegetation index values (NDVI, a “greenness” index) drawn from the Advanced Very High Resolution Radiometer (AVHRR) satellite (http://daac.gsfc.nasa.gov/avhrr/) (1982-1992). All environmental databases used in our analyzes covered areas of accessibility for each of the rodent species (see above), resampled to a spatial resolution of 2.5’ (~5 km).

### Ecological niche models

Ecological niche models were produced using Maxent version 3.2.1 [50]. We used a random seed to generate 10 replicate analyses based on bootstrap subsampling. We used median output grids as a hypothesis of suitability, and imported the results into ArcView 3.3 for assessment and analysis.

Distributional data for *N. lasiurus* and *O. nigripes* were separated into two sets: one for model calibration (75% of points) and one for model evaluation (25% of points). For infected rodents, in light of smaller sample sizes, we considered all points in the analysis. Raw Maxent outputs were converted into binary maps of suitability or unsuitability for each species based on a threshold that includes 95% of the records of each species used in model calibration [51]. This threshold takes into consideration an estimate of the likely amount of error among the occurrence data (*E* = 5%) [52]. 

### Model evaluation

We assessed model accuracy by examining omission rates associated with test points [53]. To test model significance, we compared predictive success of models against null expectations using a cumulative binomial test [34]. In particular, we assessed whether each test point fell in areas identified by the model as suitable, and compared this success rate with overall proportions of pixels identified as suitable or unsuitable for that species. Statistical significance was assessed via a cumulative binomial probability calculation in Excel. We also used Maxent’s jackknife test to identify variables that most influenced model predictions [50]. 

Finally, we used randomization tests to assess the degree to which the environmental footprint of distributions of the two putative rodent reservoirs and human cases differed. That is, if one or the other of the rodents were not involved in transmission of the virus to humans, its ecological niche might differ from that of human cases. We used the background similarity tests of Warren et al. [54], on the basis that these tests allow specification of an accessible area (**M**) particular to each of the species involved, which is key in erecting appropriate tests [55]. Specifically, we compared ecological niche model outputs thresholded at minimum training presence in terms of the similarity indices *D* and *I* [54], and compared these observed similarities to a distribution of ‘background’ similarities, in which one species’ occurrences were replaced by a similar number of random points drawn from across its M. All resampling and similarity calculations were performed in ENMTools version 1.3 [56]. Smaller numbers of variables were generated for this analysis through a principal component analysis (PCA) of the environmental variables used for niche modeling using NicheA version 1.2 [57]. For a visualization of rodent and human cases environmental use, niches were determined by a minimum-volume ellipsoid calculated around occurrences with a strict threshold (*E* = 10%; H. Qiao, pers. comm.).

## Results

**Figure 1:**
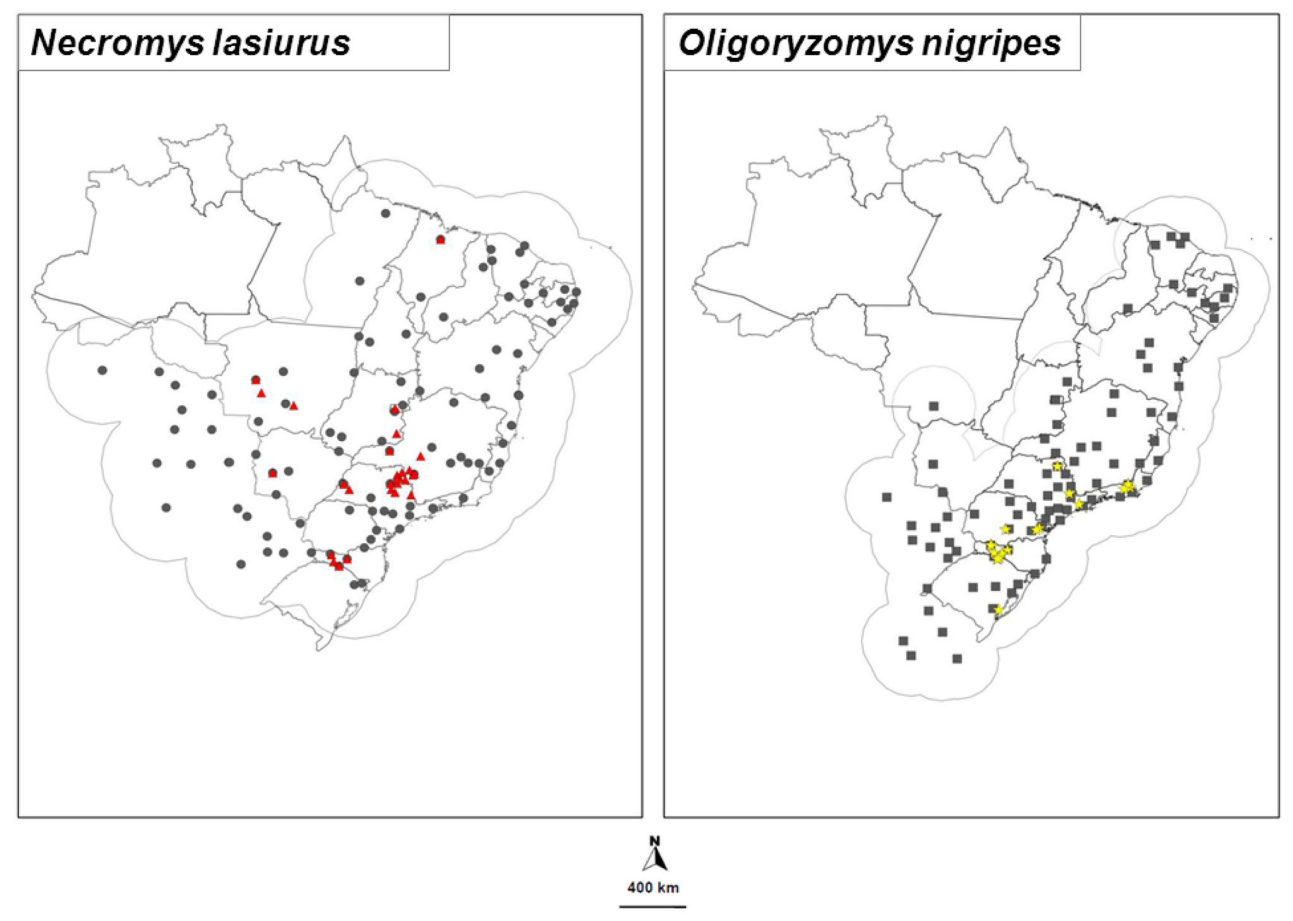
Distribution of rodent species. *Necromys lasiurus* (circles) and *Oligoryzomys nigripes* (squares). The access area "M" is approximated by buffers of 400 km. Distribution of hantavirus-infected rodents (*N. lasiurus*, red triangles and *O. nigripes*, yellow stars) is also shown.

We obtained 114 records for *N. lasiurus* and 30 records of hantavirus-infected *N. lasiurus* (Figure 1). For *O. nigripes*, we found 105 unique records and 19 records of hantavirus-infected *O. nigripes* (Figure 1). 

Niche models generated for rodents were statistically robust. For *N. lasiurus*, only one test point fell outside the predicted area of presence (3% omission error), such that binomial tests indicated statistical significance (*p* = 0.02). In *O. nigripes* comparisons, all test points were included in the predicted suitable area (0% omission), and models were thus highly statistically significant (*p* < 0.01). 

**Figure 2:**
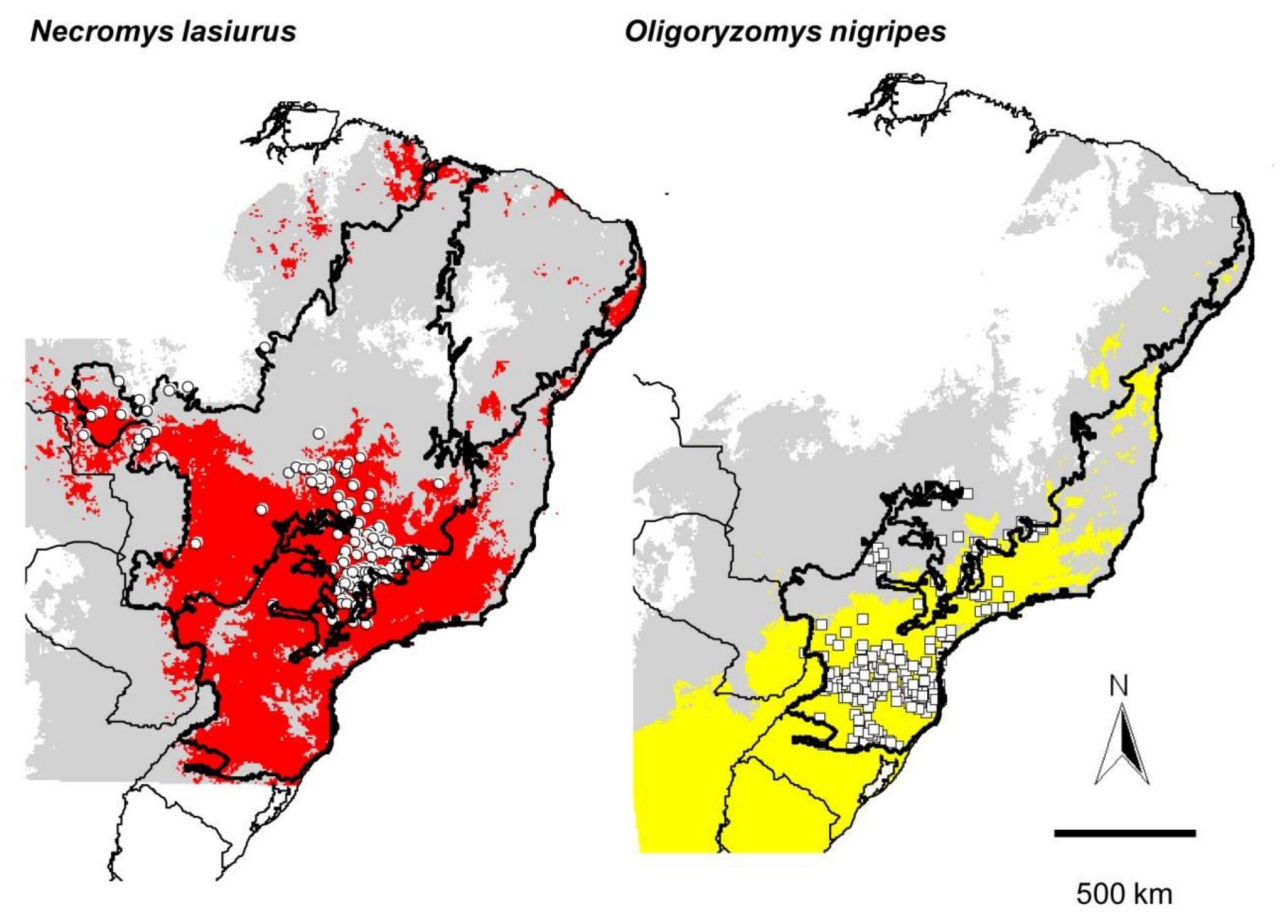
Ecological niche models projected as potential distributions for rodent reservoirs of hantavirus in Brazil (gray shading). *Necromys lasiurus* (left) and *Oligoryzomys nigripes* (right). Areas identified as suitable based on all occurrence records are shown in pale gray, whereas areas identified as suitable based only on infected rodents database are shown for *N. lasiurus* (red) and *O. nigripes* (yellow). Bold lines indicate the range of the Cerrado and Atlantic Forest. Cases of human infections with hantavirus in Cerrado (circles) and Atlantic Forest (squares) were superimposed on the models.

When niche models were displayed in geographic space *N. lasiurus* showed a wide potential distribution in Brazil. Highest climate suitability for *O. nigripes* was observed along the Brazilian Atlantic coast (Figure 2). 

When contribution of environmental variables was explored, maximum temperature in the warmest months was the variable that most influenced models of *N. lasiurus*; NDVI had low contribution. For *O. nigripes* annual precipitation was the variable that most influenced models, and NDVI data showed low contribution. 

Models based on occurrences of infected rodents estimated a broad area of hantavirus transmission in southeastern and southern Brazil, coinciding with the distribution of 280 and 188 human cases of HCPS in the Cerrado and Atlantic Forest respectively (Figure 2). Parts of northern and northeastern areas of Brazil were also suitable for occurrence of *N. lasiurus* and *O. nigripes* (Figure 2), yet show few human HCPS reports.

**Figure 3:**
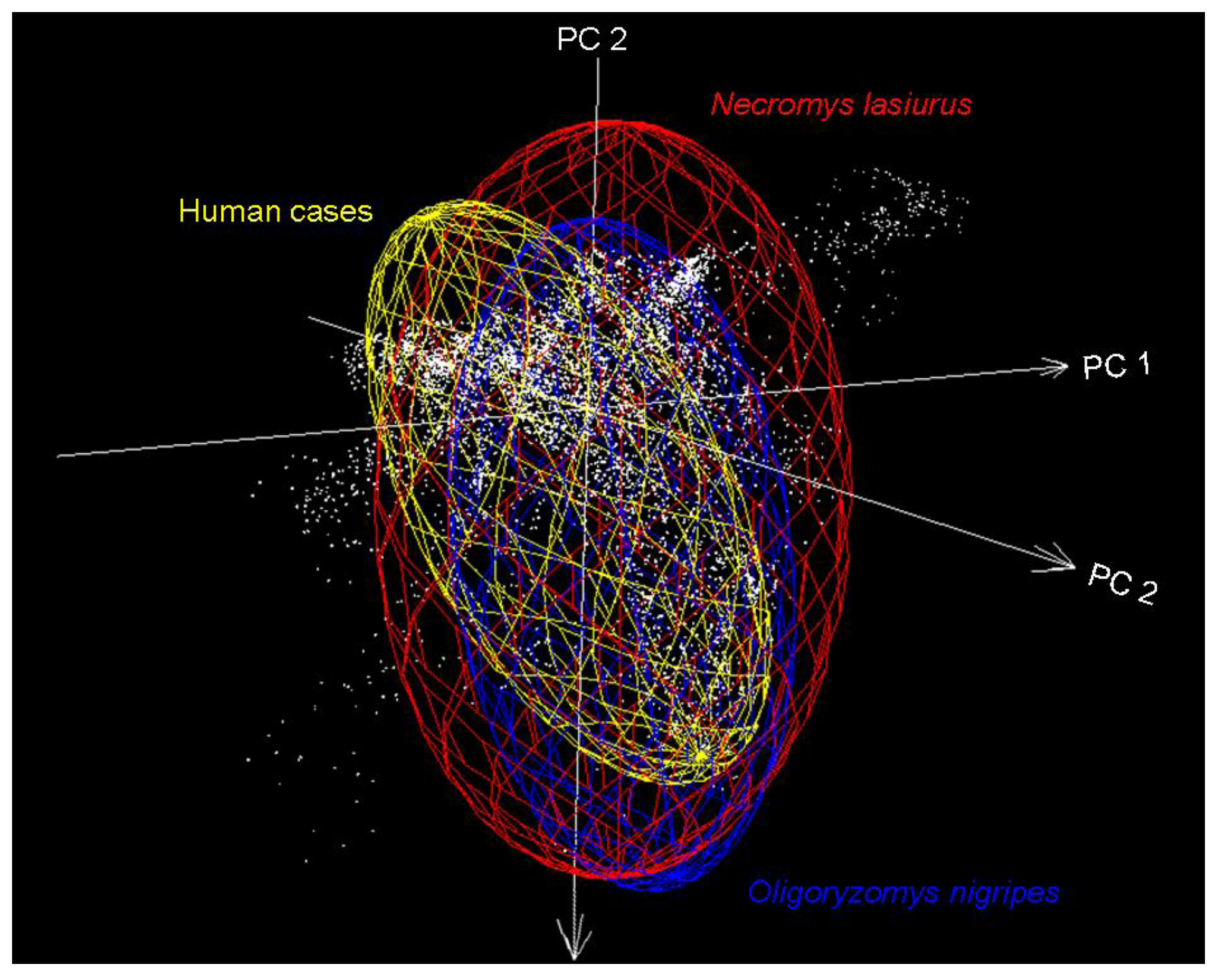
Visualization of niches of hantavirus in rodents and humans. White points indicate environments in the study area (background). Ellipsoids are the representation of niches for hantavirus in *N. lasiurus* (red), *O. nigripes* (blue), and human cases (yellow). Environmental space based on components 1 to 3 from a principal component analysis (PCA) of climatic and NDVI variables. Note that, while the ellipsoids for the different species do show some difference, so also do the environments represented within each species’ **M**; here, the results of the background similarity test become key.

Background similarity tests, in all comparisons, failed to reject the hypothesis of niche similarity (Table 1). That is, observed similarity values fell within the null distribution of background similarity values. In this sense, no evidence was available that suggested that these two rodent species could not be involved in hantavirus transmission. The first three axes from PCA analyses explained 90% of the variance; visualization of niches in the environmental space allows appreciation of overlap between rodent species and human cases (Figure 3), this overlap corroborated our similarity test results.

**Table 1:** Background similarity test results for two similarity metrics (*I* and *D*) for comparisons of *Necromys lasiurus* and *Oligoryzomys nigripes* (putative reservoirs) and human cases.

		**Observed overlap**			***I***			***D***	
**Target versus background**		*I*	*D*		5%	95%		5%	95%
Human cases versus *Necromys lasiurus* background		0.895	0.849		0.861	0.947		0.809	0.929
Human cases versus *Oligoryzomys nigripes* background		0.925	0.909		0.869	0.944		0.811	0.929
*Necromys lasiurus* versus *Oligoryzomys nigripes* background		0.918	0.856		0.846	0.919		0.834	0.867
*Oligoryzomys nigripes* versus *Necromys lasiurus* background		0.918	0.856		0.882	0.961		0.840	0.940

Observed overlap values can be compared against the 5% (dissimilar) and 95% (similar) values for the null distributions. As can be appreciated, all observed values fell within null expectations; i.e., the hypothesis of niche similarity could not be rejected.

## Discussion

This study updates the picture of the geographic distribution of *N. lasiurus*. Bonvicino et al. [6] showed only limited occurrence of *N. lasiurus* in the Atlantic Forest biome. We found records of *N. lasiurus* in many municipalities of Atlantic Forest, albeit largely on the interior slopes of the coastal mountain ranges. These occurrences could be favored by presence of *Brachiaria* spp. as has been suggested [7,58], in open areas or near remnants of Atlantic Forest. *N. lasiurus* can be found in many habitats, but prefer open and dry areas, being absent or infrequent in moist forest environments [59,60]. Occurrence of *N. lasiurus* has also been observed in areas of agricultural and urban expansion [58], suggesting that deforestation over recent years favors occurrence of the species in the Atlantic Forest biome. The reproduction of *N. lasiurus* occurs throughout the year with seasonal peaks. Litter sizes of these species are similar, varying from 1 to 13 new individuals [61]. Annual population fluctuations appear to be regulated by food availability, influenced by rainfall [62].

Our results also extend the limits of the distribution of *O. nigripes* in Brazil, including areas in the extreme south (Rio Grande do Sul), northeast (Ceará) and Midwest (Mato Grosso and Mato Grosso do Sul) of the country. Predictive models indicated favorable conditions for occurrence of the species outside the limits of the Atlantic Forest within the Cerrado and Pantanal biomes. In these areas, *O. nigripes* is associated with gallery forest, secondary forest, and forest edges [63].

Our model results agree with analyses carried out in Argentina by Carbajo & Pardiñas (2007) [8], indicating that aspects of both temperature and precipitation were essential to explain the distribution of *O. longicaudatus*. Those authors also found an association between occurrences of human hantavirus cases in areas with a higher suitability for *O. longicaudatus*. 

Recently, Donalísio & Peterson [10], seeking to identify environmental factors influencing occurrence of hantavirus cases in southern Brazil, found that winter precipitation and high photosynthetic mass were most closely related to distributions of cases of HCPS; the authors explored distributions of four species of *Oligoryzomys* in relation to areas of hantavirus transmission in southern Brazil. NDVI showed low contribution to our models, in contrast with results of Donalísio & Peterson [10]. 

We found a broad potential distributional area for infected rodents in Mato Grosso do Sul, in the southwestern Cerrado region, where only one HPCS case was registered. Low reporting owing to diagnostics by local health services may be involved in the low incidence of HPCS in the predicted area. Future studies should evaluate rates of infection in rodents, using records collected via careful planned sampling in areas with lack of information but with suitable environments predicted by our models in order to fill epidemiological gaps. 

Most HCPS cases in Brazil (85%) occurred within the Cerrado and Atlantic Forest biomes. These biomes cover 37% of the area of Brazil, where ~140 million people live, representing 73% of the population of the country (http://www.ibge.gov.br). Our exclusion of the Amazon Basin from analyses was owing to unavailability of information on reservoirs and their occurrences. Recent studies in Mato Grosso showed that *Oligoryzomys utiaritensis* [64] is involved in hantavirus transmission to humans [65]; indeed, studies showing involvement of *Calomys callidus* [66] and *O. fornesi* in transmission [66] indicate that these species are also involved in the virus cycle in Brazil.

Considering the high rodent species richness in the Cerrado (78 species) and Atlantic Forest (98 species) [67] biomes, with little knowledge of enzootic cycles of hantavirus in these biomes, it is clear that we are still far from fully understanding the natural history of hantaviruses in Brazil. Our ecological niche models for *N. lasiurus* and *O. nigripes* indicate that climatic variables are fundamental to explaining distributions of these species, at least over the broad extent of this study. At the spatial scale explored, niches of *N. lasiurus* and *O. nigripes*, and human hantavirus reports were not significantly different, highlighting a role for *N. lasiurus* and *O. nigripes*, at least in part, in the host-parasite cycle of hantavirus and eventual transmission to humans. We stress the importance of knowledge of true participation of these rodents in epidemiological cycles of the disease, because the predictive model of infected rodents included sites of HCPS cases in Cerrado and Atlantic Forest. 
